# Neuropilins guide preganglionic sympathetic axons and chromaffin cell precursors to establish the adrenal medulla

**DOI:** 10.1242/dev.162552

**Published:** 2018-11-02

**Authors:** Rachael Lumb, Mathew Tata, Xiangjun Xu, Andrew Joyce, Ceilidh Marchant, Natasha Harvey, Christiana Ruhrberg, Quenten Schwarz

**Affiliations:** 1 Centre for Cancer Biology, SA Pathology and University of South Australia, North Terrace, Adelaide 5001, Australia; 2 Medical School, University of Adelaide, Frome Road, Adelaide 5000, Australia; 3 UCL Institute of Ophthalmology, University College London, 11-43 Bath Street, London EC1V 9EL, UK

**Keywords:** Adrenal gland, Autonomic nervous system, Axon guidance, Chromaffin cell, Neural crest cell, Neuropilin

## Abstract

The adrenal medulla is composed of neuroendocrine chromaffin cells that secrete adrenaline into the systemic circulation to maintain physiological homeostasis and enable the autonomic stress response. How chromaffin cell precursors colonise the adrenal medulla and how they become connected to central nervous system-derived preganglionic sympathetic neurons remain largely unknown. By combining lineage tracing, gene expression studies, genetic ablation and the analysis of mouse mutants, we demonstrate that preganglionic axons direct chromaffin cell precursors into the adrenal primordia. We further show that preganglionic axons and chromaffin cell precursors require class 3 semaphorin (SEMA3) signalling through neuropilins (NRP) to target the adrenal medulla. Thus, SEMA3 proteins serve as guidance cues to control formation of the adrenal neuroendocrine system by establishing appropriate connections between preganglionic neurons and adrenal chromaffin cells that regulate the autonomic stress response.

## Abstract (precis)

**Summary:** A new role is revealed for semaphorin/neuropilin signalling in guiding preganglionic sympathetic axons and chromaffin cell precursors into the adrenal primordia, ensuring correct regulation of the autonomic stress response.

## INTRODUCTION

Neuroendocrine chromaffin cells of the adrenal medulla produce adrenaline and noradrenaline to regulate the function of internal organs for physiological homeostasis and to initiate the ‘fight-or-flight’ stress response (Lumb and Schwarz, 2015). Paramount to correct chromaffin cell function is cholinergic input from preganglionic neurons whose cell bodies are located within the central nervous system and which project axons to the adrenal medulla to form synapses with chromaffin cells. Surprisingly, it remains unknown how connections between preganglionic neurons and chromaffin cells are established.

Together with sympathetic neurons of the autonomic nervous system, chromaffin cells of the adrenal medulla originate from a common sympathoadrenal neural crest cell (NCC) precursor that splits between sympathetic and chromaffin lineages after completion of NCC migration (Furlan et al., 2017). Sympathoadrenal NCCs delaminate from dorsal neural folds at somite levels 18-24 and migrate towards the dorsal aorta under instruction of several receptor-ligand pairs (Le Douarin and Kalcheim, 1999), including SDF1 and its receptor CXCR4 (Britsch et al., 1998), neuregulin and its receptors ERBB2 and ERBB3 (Britsch et al., 1998), and class 3 semaphorins (SEMA3s) and their receptors NRP1 and NRP2 (Lumb et al., 2014; Schwarz et al., 2009b). However, contrasting models have been put forward to explain how a subset of sympathoadrenal NCCs navigates into the anlagen of the adrenal primordia. In the first model, BMPs secreted from the dorsal aorta have been proposed to induce expression of neuregulin 1 (NRG1) and stromal cell-derived factor 1 (SDF1) within the para-aortic mesenchyme to act directly on the chromaffin cell precursors to promote their migration towards the adrenal primordia (Saito et al., 2012). In a competing model, a proportion of chromaffin cells has been suggested to arise from sympathoadrenal NCCs that first transition through an intermediate Schwann cell precursor (SCP) state: these SCPs are proposed to migrate along preganglionic sympathetic nerve fibres into the adrenal medulla, where they differentiate into chromaffin cells (Furlan et al., 2017).

Here, we have combined lineage tracing, gene expression studies, cell-specific genetic ablation and the analysis of mouse knockouts to show that chromaffin cell precursors require preganglionic axons to colonise the adrenal primordia. We further identify semaphorin signalling through NRP1 and NRP2 as an important signalling pathway that guides axons and chromaffin cell precursors into this tissue. Although NRP2 is required for preganglionic axonal guidance into the adrenal primordia, NRP1 is required cell-autonomously for chromaffin cell precursors to maintain their association with the axon scaffold. Taken together, our data are consistent with the model in which chromaffin cells arise from SCPs that migrate into the adrenal primordia along preganglionic sympathetic nerve fibres. This work therefore identifies a new molecular mechanism connecting the central and peripheral components of the autonomic nervous system that regulate the ‘flight-or-flight’ stress response.

## RESULTS

### Chromaffin cell precursors are tightly associated with preganglionic axons

**Fig. 1.**
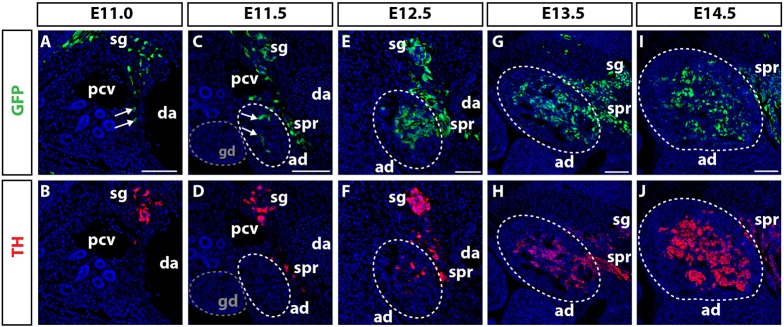
**NCC colonisation of the adrenal primordia.** (A-J) Transverse sections through E11.0-E14.5 *Wnt1-Cre; Z/EG* embryos immunolabelled for GFP and TH. (A,B) At E11.0, a small number of GFP^+^ NCCs have migrated ventrally from the sympathetic ganglia (sg) and sit between the posterior cardinal vein (pcv) and the dorsal aorta (da) (arrows). (C,D) At E11.5, the adrenal primordia (ad, white dashed circle) splits from the gonadal tissue (gd, grey dashed circle). GFP^+^ NCCs are observed in the suprarenal ganglia (spr) and have begun to colonise the adrenal primordia (arrows). (E,F) At E12.5, the number of NCCs within the adrenal primordia has expanded with a small number of these beginning to express TH. (G,H) At E13.5, the majority of NCCs in the adrenal primordia express TH. (I,J) By E14.5 NCC-derived chromaffin cells have begun to condense into the mature medulla. Scale bars: 100 µm.

**Fig. 2.**
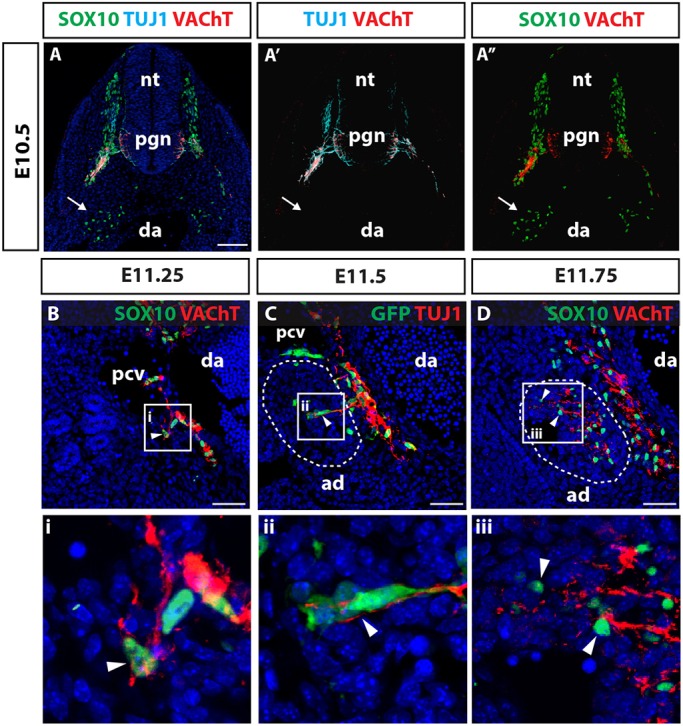
**Preganglionic axons innervate the adrenal primordia in unison with NCC colonisation.** (A-A″) Transverse sections through somites 18-24 of an E10.5 wild-type embryo immunolabelled for TUJ1, VAChT and SOX10 demonstrates SOX10^+^ NCCs reach the dorsal aorta (da) to seed the sympathetic ganglia (white arrow) prior to innervation of this region by VAChT^+^ preganglionic neurons (pgn) located in the ventral neural tube (nt). (B-D) Transverse sections through E11.25-E11.75 wild-type (B,D) and *Wnt1-Cre; Z/EG* (C) embryos immunolabelled for axonal and NCC markers. (B) Preganglionic axons track ventrally from the sympathetic ganglia between the posterior cardinal vein (pcv) and dorsal aorta, and begin to sprout laterally toward the prospective adrenal primordia (ad) in close association with SOX10^+^ NCCs (arrowhead). (C) As the adrenal primordia emerges from the adrenogonadal precursor, axons sprout laterally into the primordial tissue, concomitant with NCC colonisation (arrowhead). (D) At E11.75, preganglionic axons arborise within the adrenal primordia aligned with NCCs (arrowheads). Blue, DAPI. Scale bars: 100 µm.

Preganglionic neurons that connect to the adrenal medulla are thought to reside in the intermediolateral motor column of the spinal cord (Appel and Elde, 1988). To establish the position of these neurons within the central nervous system, we implanted DiI into the adrenal primordia of E12.5 embryos. Retrograde tracing identified cell bodies in the intermediolateral motor columns of the neural tube, similar to immunostaining for the cholinergic marker vesicular acetylcholine transporter (VAChT) (Fig. S1). To define the time at which NCCs and preganglionic axons colonise the adrenal primordia, we lineage-traced NCCs and their derivatives by using *Wnt1-Cre* mice crossed to the *Z/EG* reporter line. GFP^+^ NCCs entered the adrenal primordia between E11.0 and E11.5, and began to differentiate into tyrosine hydroxylase (TH)^+^ chromaffin cells 24 h later (Fig. 1A-F, arrows). The number of GFP^+^ cells within the adrenal primordia expanded up until E14.5, with many cells expressing TH (Fig. 1G-J). Immunolabelling for the pan-neuronal TUJ1 antibody and the cholinergic VAChT antibody identified pioneering preganglionic axons sprouting into the prospective adrenal primordia in close association with GFP^+^ and SOX10^+^ NCCs between E11.25 and E11.5 (Fig. 2B-D, arrowheads). In contrast, the migration of sympathoadrenal NCCs toward the dorsal aorta at earlier developmental time points occurred in the absence of preganglionic axons (Fig. 2A, arrow). Taken together, this analysis demonstrates that chromaffin cell precursors associate with preganglionic axons and that both cell types colonise the adrenal primordia together between E11.25 and E11.5.

### Chromaffin cell precursors require preganglionic axons to migrate into the adrenal primordia

**Fig. 3.**
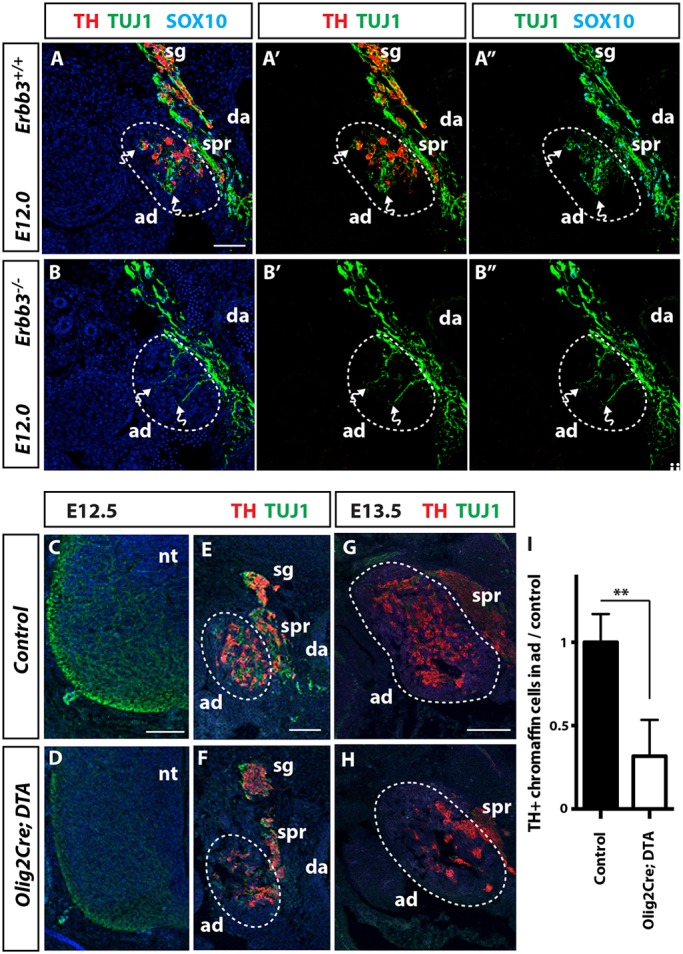
**Preganglionic axons guide chromaffin cell precursors into the adrenal medulla.** (A-B″) Transverse sections through E12.0 *Erbb3^+/+^* (A-A″) and *Erbb3^−/−^* (B-B″) embryos immunolabelled for SOX10, TUJ1 and TH. Axons sprout laterally into the adrenal primordia of *Erbb3^−/−^* embryos that lack all sympathoadrenal NCCs (bent arrows). (C-H) Transverse sections through E12.5-E13.5 wild-type and *Olig2Cre; DTA* embryos immunolabelled for TUJ1 and TH. TUJ1 staining confirms ablation of neurons in the ventral neural tube (nt) of *Olig2Cre; DTA* embryos (C,D). (I) Quantitation of TH staining shows a significant reduction of chromaffin cells in the adrenal primordia (ad) of E12.5 *Olig2Cre; DTA* embryos (*n*=5/genotype). sg, sympathetic ganglia; da, dorsal aorta; spr, suprarenal ganglia; blue, DAPI. Data are mean±s.e.m. and analysed using two-tailed Student's *t*-test; **P<0.005. Scale bars: 100 µm.

Synchronised entry of preganglionic axons and chromaffin cell precursors into the adrenal primordia raised the hypothesis that these distinct cell types may cooperate with each other to colonise this tissue. To address whether axon innervation depends on chromaffin cell precursors, we analysed *Erbb3^−/−^* embryos that lack all sympathoadrenal NCCs and their derivatives (Britsch et al., 1998). As the absence of sympathoadrenal NCCs did not prevent axons entering the adrenal primordia (Fig. 3A-B), our data demonstrate that axons do not require chromaffin cell precursors to innervate this organ. To determine whether chromaffin cell precursors instead depend on axonal innervation, we ablated preganglionic neurons by crossing *Olig2-Cre* mice to *Rosa^DTA^* mice. In these mice, the cell-lethal diphtheria toxin A (DTA) is activated in the progenitors of motoneurons and oligodendrocytes in the ventral spinal cord by CRE expressed under control of the *Olig2* promoter (Wu et al., 2006). Analysis of the resulting embryos was performed at E12.5 when motoneurons have formed, but not oligodendrocytes, which differentiate only after motoneuron generation is complete (Wu et al., 2006). Ablation of motoneurons and their axons (Fig. 3C-F) lead to a 69% reduction of chromaffin cells in the adrenal glands (Fig. 3E,F,I; *n*=5/genotype; *P*=0.001). This reduction is unlikely to result from a developmental delay, because chromaffin cells were also deficient in *Olig2-Cre; Rosa^DTA^* mice at E13.5 (Fig. 3G,H). Consistent with previous findings using *HB9-Cre* mice to ablate preganglionic neurons (Furlan et al., 2017), our data show that NCC-derived chromaffin cell precursors require preganglionic axons to colonise the adrenal primordia.

### NRPs are differentially expressed on preganglionic axons and chromaffin cell precursors

**Fig. 4.**
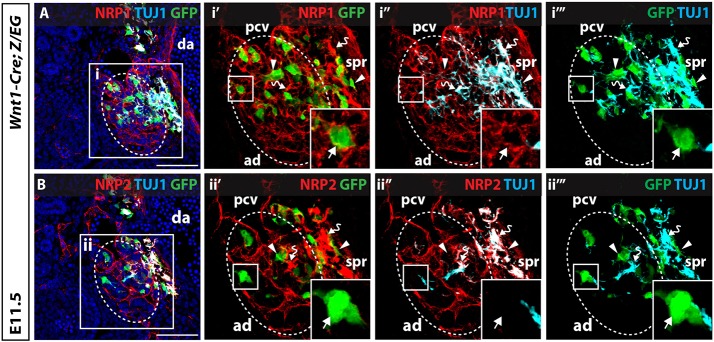
**Differential expression of NRPs in sympathoadrenal NCCs and preganglionic axons.** (A,B) Serial transverse sections through E11.5 *Wnt1-Cre; Z/EG* embryos immunolabelled for GFP, NRP1 and TUJ1 (A) and GFP, NRP2 and TUJ1 (B). Insets in i′-i‴,ii′-ii‴ show higher magnification of boxed areas. NRP1 and NRP2 are expressed on axons arborising within the adrenal primordia (ad; bent arrows) and on axons tracking toward the adrenal primordia and suprarenal ganglia (spr). NRP2 is also present on blood vessels within the adrenal primordia. Axons innervating the adrenal primordia are tightly associated with GFP^+^ NCCs (white arrowheads). NRP1, but not NRP2, is expressed on NCCs that have migrated into the adrenal primordia (white arrows). pcv, posterior cardinal vein; da, dorsal aorta; blue, DAPI. Scale bars: 100 µm.

Because the presence of preganglionic axons is essential for chromaffin cell precursors to reach the adrenal primordia, we next asked which guidance factors are required for preganglionic axon navigation. NRG1 is expressed within the region of the prospective adrenal primordia and required for chromaffin cell colonisation of the adrenal medulla (Saito et al., 2012). To investigate whether NRG1 may also be required for axonal innervation, we analysed *Erbb3^−/−^* mice, which lack the NRG1 receptor ERBB3, but observed normal axonal innervation of the adrenal tissue (Fig. 3A-B). We next examined whether NRP1 and NRP2 play a role, because they are receptors for the axon guidance cues SEMA3A and SEMA3F (Schwarz et al., 2009a,b), and make up part of the genetic network that distinguishes differentiated chromaffin cells from their precursors (Furlan et al., 2017). Consistent with this notion, lineage tracing of NCCs and their derivatives in *Wnt1-Cre;Z/EG* embryos demonstrated that chromaffin cell precursors, which were positioned along axons innervating the adrenal primordia, expressed NRP1 on their surface, but lacked NRP2 (Fig. 4A,B and Fig. S2A-C, arrows). In contrast, NRP2 expression at the ventral boundary of the adrenal primordia marked cells that were also positive for TH and had begun to differentiate into sympathetic neurons or chromaffin cells (Fig. 4B and Fig. S2D-I, open arrow). Immunostaining of GFP^+^ NCCs isolated from the aorta-gonad-mesonephros region of E11.5 *Wnt1-Cre;Z/EG* embryos further confirmed that all SOX10^+^ chromaffin cell precursors express NRP1 on their cell surface (Fig. S3; *n*=123/123 GFP^+^ cells). Notably, NRP1 and NRP2 were both present on preganglionic axons innervating the adrenal primordia (Fig. 4A,B, curved arrows), with NRP2 also expressed on blood vessels within the adrenal tissue (Fig. 4B). The NRP1 and NRP2 ligands *Sema3a* and *Sema3f* were expressed in connective tissue surrounding the preganglionic axons innervating the sympathetic chain and adrenal primordia (Fig. S4). Together, these expression patterns raised the possibility that preganglionic axons use NRP1 and NRP2 for their pathfinding into the adrenal primordia, and that chromaffin cell precursors use NRP1 to navigate into the adrenal primordia.

### NRP2 directs preganglionic axons into the adrenal primordia

**Fig. 5.**
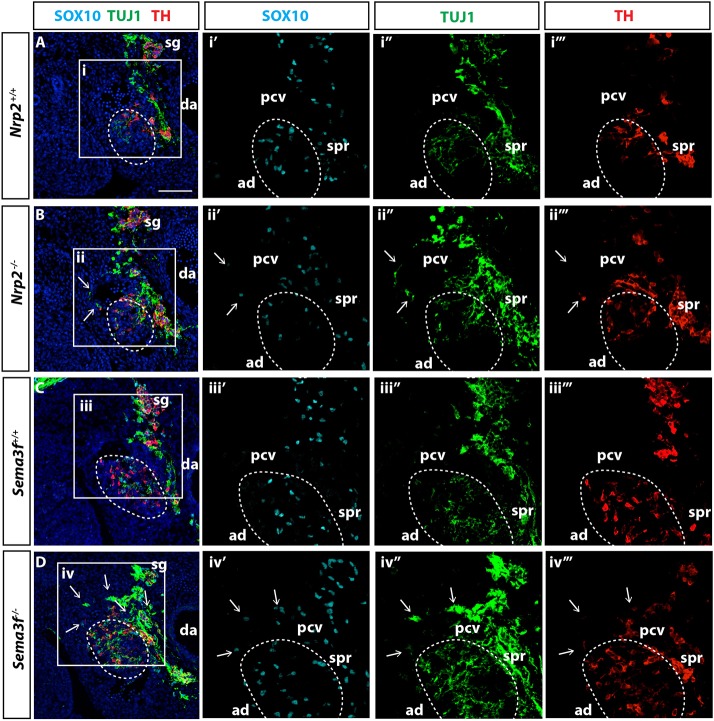
**NRP2 guides preganglionic axons into the adrenal primordia.** (A-D) Transverse sections through the adrenal primordia of E12.5 *Nrp2^+/+^* (A), *Nrp2^−/−^* (B), *Sema3f^+/+^* (C) and *Sema3f^−/−^* (D) embryos immunolabelled for SOX10, TUJ1 and TH. (B) Preganglionic axons are misguided in *Nrp2^−/−^* embryos. Axons aberrantly navigate around the posterior cardinal vein (pcv) with SOX10^+^ chromaffin cell precursors and TH^+^/TUJ1^low^ chromaffin cells in tight association (arrows). (D) Axons are misguided around the posterior cardinal vein in *Sema3f^−/−^* embryos and aligned with SOX10^+^ NCCs and TH^+^/TUJ1^low^ chromaffin cells (arrows). ad, adrenal primordia; da, dorsal aorta; spr, suprarenal ganglia; blue, DAPI; *n*=5/genotype. Scale bar: 100 µm.

To determine whether SEMA3F signalling through NRP2 regulates preganglionic axon guidance, we examined *Nrp2^−/−^* and *Sema3f^−/−^* embryos. In both types of mutants, we observed ectopic TUJ1/VAChT^+^ preganglionic axons that had extended laterally around the posterior cardinal vein (Fig. 5A-C and Fig. S5) and had aberrantly innervated the urogenital ridge (Fig. S6). In all cases examined, chromaffin cell precursors (SOX10^+^ TH^−^) and chromaffin cells (TH^+^ TUJ1^low^) were present alongside the misguided axons (Fig. 5, Figs S5 and S6, open arrows). NRP2 is therefore necessary to guide preganglionic sympathetic axons and associated chromaffin cell precursors into the adrenal primordia.

### NRP1 controls chromaffin cell precursor migration cell autonomously

**Fig. 6.**
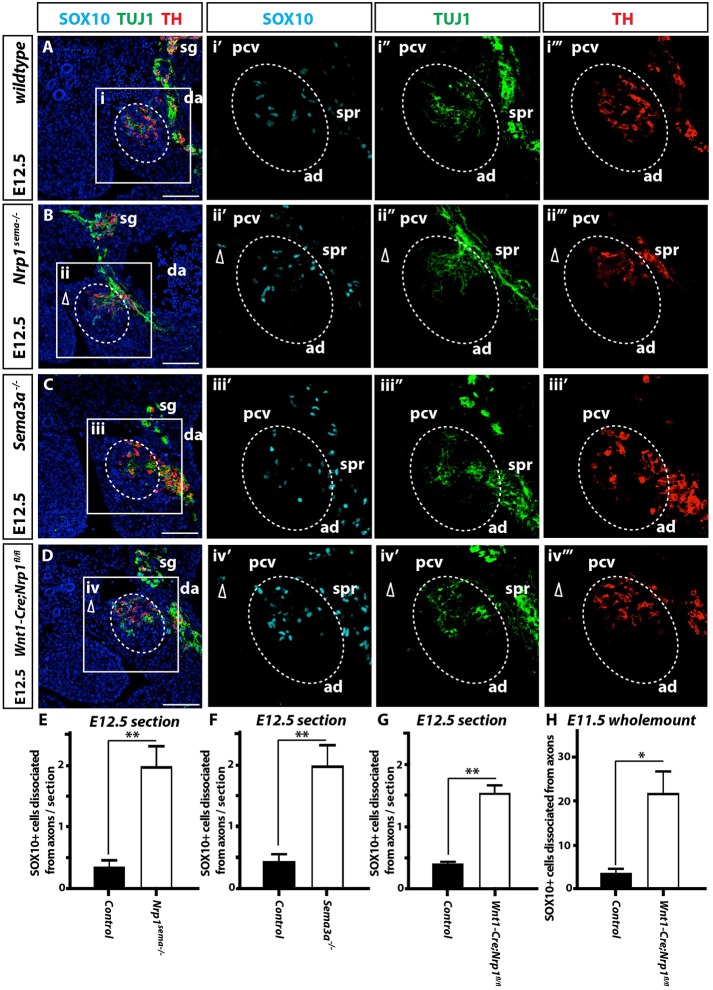
**NRP1 controls sympathoadrenal NCC migration cell autonomously.** Transverse sections through the adrenal primordia (ad) of E12.5 control (A), *Nrp1^sema−/−^* (B), *Sema3a^−/−^* (C) and *Wnt1-Cre; Nrp1^fl/fl^* (D) embryos immunolabelled for SOX10, TUJ1 and TH. Preganglionic axons track through the sympathetic ganglia (sg) and between the dorsal aorta (da) and posterior cardinal vein (pcv) before innervating the adrenal primordia and suprarenal ganglia (spr). Axons innervate the adrenal ganglia and suprarenal ganglia normally in *Nrp1^sema−/−^* (B), *Sema3a^−/−^* (C) and *Wnt1-Cre; Nrp1^fl/fl^* (D) embryos. (B) *Nrp1^sema−/−^* mice had ectopic SOX10^+^ NCCs near the posterior cardinal vein (pcv) (open arrowhead). (C) No ectopic SOX10^+^ NCCs were identified around the adrenal primordia of *Sema3a^−/−^* embryos. (D) *Wnt1-Cre; Nrp1^fl/fl^* embryos had ectopic SOX10^+^ NCCs near the posterior cardinal vein (open arrowhead). (E-G) Quantitation of SOX10^+^ cells dissociated from axons/section of E12.5 *Nrp1^sema−/−^*, *Sema3a^−/−^* and *Wnt1-Cre; Nrp1^fl/fl^* mutants compared with littermate controls; *n*=5/genotype; **P<0.005. (H) Quantitation of SOX10^+^ cells dissociated from axons around the adrenal primordia of E11.5 *Wnt1-Cre; Nrp1^fl/fl^* mutants; *n*=5/genotype; *P<0.05. Blue, DAPI. Data are mean±s.e.m. and were analysed using two-tailed Student's *t*-test. Scale bars: 100 µm.

To determine whether SEMA3A signalling through NRP1 also regulates preganglionic axon guidance, we next analysed *Sema3a^−/−^* mutants and *Nrp1^sema−/−^* mutants, which carry a seven amino acid substitution that compromises SEMA3 binding to NRP1 (Gu et al., 2003). In contrast to the profound defasciculation of axons tracking to the forelimb, preganglionic axons were largely unaffected in *Sema3a^−/−^* and *Nrp1^sema−/−^* embryos (Fig. 6A-C). Nevertheless, we also observed ectopically positioned SOX10^+^ NCCs and TH^+^ TUJ1^low^ adrenal chromaffin cells adjacent to the adrenal primordia (Fig. 6A-C,E,F, open arrowheads; *n*=5/5 for both types of mutants) and within the urogenital ridge in areas devoid of ectopic axons (Fig. S7A-C, open arrowheads; *n*=5/5 for both types of mutants). These findings suggest that SEMA3A signalling through NRP1 contributes to chromaffin cell precursor guidance, independently of a role in guiding the axons they are associated with. To explore whether NRP1 has a cell-autonomous role in chromaffin cell precursor migration, we examined *Wnt1-Cre; Nrp1^fl/fl^* mutants, which lack NRP1 in the NCC lineage but retain NRP1 in the preganglionic axons that innervate the adrenal primordia, because their cell bodies are located in the ventral spinal cord, which is not targeted by *Wnt1-Cre*. We observed ectopic SOX10^+^ NCCs flanking the adrenal primordia (Fig. 6D,G, open arrowhead; *n*=5/5 mutants), and ectopic chromaffin cells in the urogenital ridge (Fig. S7D, open arrowhead; *n*=5/5 mutants). Analysis of whole E11.5 embryos further demonstrates that ectopic SOX10^+^ NCCs are dissociated from both TUJ1^+^ and TH^+^ axons (Fig. S8 and Fig. 6H; *n*=5/5 mutants). To investigate whether SEMA3A acts directly on chromaffin cell precursors, we treated GFP^+^ NCCs isolated from the aorta-gonad-mesenephros region of E11.5 *Wnt1-Cre;Z/EG* embryos with recombinant SEMA3A. After addition of SEMA3A, cellular protrusions collapsed, with retraction of both filopodia and lamellipodia (Fig. S9, *n*=36 GFP^+^ NCCs). SEM3A/NRP1 signalling therefore regulates the association of chromaffin cell precursors with preganglionic sympathetic axons that target the adrenal medulla.

### NRPs cooperatively guide preganglionic axons and chromaffin cell precursors into the adrenal primordia

**Fig. 7.**
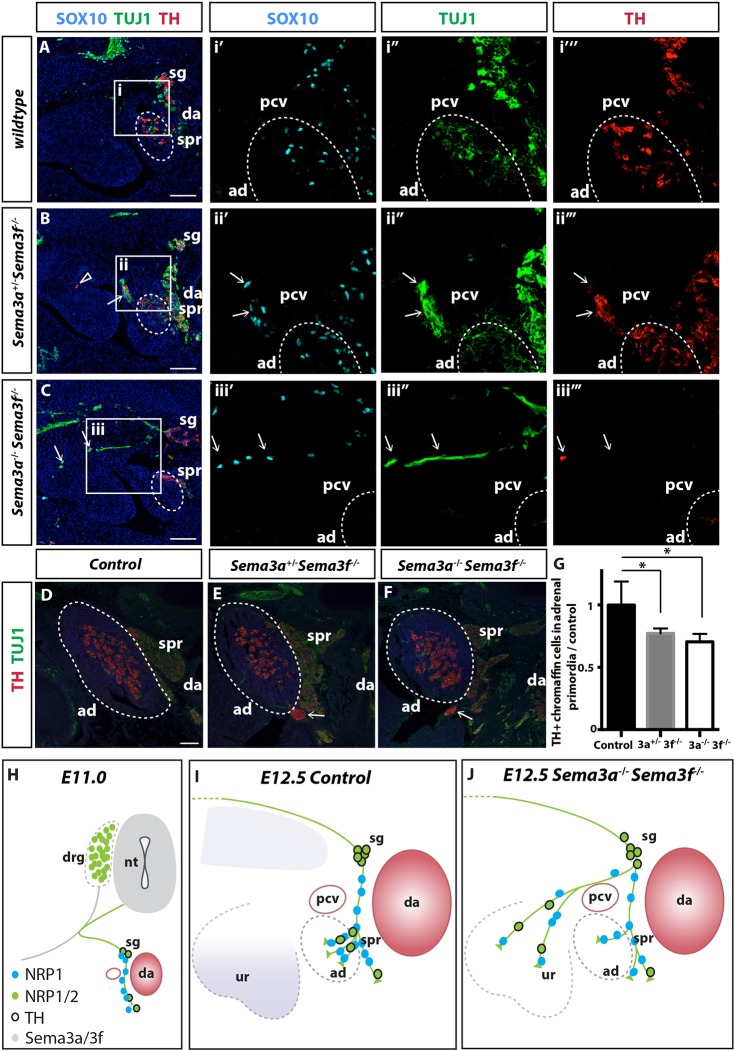
**NRPs cooperate to guide preganglionic axons and chromaffin cell precursors into the adrenal primordia.** Transverse sections through the adrenal primordia (ad) of E12.5 wild-type (A), *Sema3a^+/−^; Sema3f^−/−^* (B) and *Sema3a^−/−^; Sema3f^−/−^* (C) embryos immunolabelled for SOX10, TUJ1 and TH. (B) A large number of preganglionic axons, chromaffin cell precursors and chromaffin cells are ectopically positioned around the posterior cardinal vein (pcv; arrow) and within the urogenital ridge (open arrowhead) of *Sema3a^+/−^; Sema3f^−/−^* embryos. (C) Large axonal bundles aligned with SOX10^+^ chromaffin cell precursors and TH^+^ chromaffin cells are found in the urogenital ridge of *Sema3a^−/−^; Sema3f^−/−^* embryos (arrows). (D-F) Transverse sections through the adrenal primordia of E14.5 control (D), *Sema3a^+/−^; Sema3f^−/−^* (E) and *Sema3a^−/−^; Sema3f^−/−^* (F) embryos immunolabelled for TUJ1 and TH. TH^+^ chromaffin cells are depleted in the adrenal primordia of *Sema3a^+/−^; Sema3f^−/−^* and *Sema3a^−/−^; Sema3f^−/−^* embryos, with large ectopic chromaffin cell condensations located ventral to the adrenal primordia (arrows). (G) Quantitation of TH^+^ chromaffin cells in the adrenal primordia of E14.5 *Sema3a* and *Sema3f* compound mutants. (H-J) Schematic of how SEMA3/NRPs guide preganglionic axons and chromaffin cell precursors into the adrenal primordia between E11.0 and E12.5. da, dorsal aorta; spr, suprarenal ganglia; blue, DAPI. Data are mean±s.e.m. and were analysed using two-tailed Student's *t*-test; *n*=4/genotype; *P<0.05. Scale bars: 100 µm.

Because NRP1 and NRP2 are co-expressed in preganglionic axons, and the *Sema3a* and *Sema3f* expression domains overlap, we next examined whether these pathways cooperate to guide axons and chromaffin cell precursors into the adrenal primordia. When *Sema3a* was reduced on a *Sema3f*-null background (*Sema3a^+/−^; Sema3f^−/−^* mutants), a large number of ectopic TUJ1/VAChT^+^ axons and TH^+^ TUJ1^low^ chromaffin cells were identified adjacent to the adrenal primordia and within the urogenital ridge at E12.5 (Fig. 7A-B and Fig. S10, open arrows). Analysis at E14.5 demonstrated a 25% reduction of TH^+^ cells in the adrenal medulla of *Sema3a^+/−^; Sema3f^−/−^* mutants, with many ectopically positioned chromaffin cells positioned next to the adrenal glands (Fig. 7D-G, open arrows). This defect was compounded in double homozygous *Sema3a^−/−^; Sema3f^−/−^* mutants, in which large axonal bundles were misrouted into the urogenital ridge and the adrenal gland was depleted of TH^+^ cells (Fig. 7C,F,G and Fig. S10, open arrows). SEMA3A/NRP1 and SEMA3F/NRP2 therefore cooperatively control axonal and chromaffin cell precursor migration into the adrenal primordia (Fig. 7H-J).

## DISCUSSION

Our data substantiate the cellular mechanisms by which chromaffin cell precursors are guided into the adrenal primordia by the preganglionic axons with which they will form functional connections (Fig. 7D-F). This concept is derived from several observations, including the findings that: (1) axonal innervation coincides with chromaffin cell precursor migration into the adrenal primordia; (2) chromaffin cell precursors are intimately associated with preganglionic axons prior to colonising the adrenal primordia; (3) chromaffin cells within the adrenal medulla are dramatically reduced in the absence of preganglionic neurons; and (4) adrenal chromaffin cells follow misguided preganglionic axons in mice with defective preganglionic axon innervation of the adrenal medulla.

Prior analysis of chromaffin cell development using SCP lineage tracing and SCP genetic ablation concluded that SCPs contributed to approximately half of all chromaffin cells, with the other half arising from earlier migrating NCCs (Furlan et al., 2017). By showing that chromaffin cell precursors are associated with preganglionic axons and express the SCP marker SOX10 after NCC migration is considered to be complete (Furlan et al., 2017), our data agree with the notion that chromaffin cells arise from SCPs. However, the proportion of chromaffin precursors that requires axons for entry into the adrenal primordia remains in question. Thus, our finding that the first wave of NCCs enters the adrenal primordia in association with pioneering preganglionic axons suggests that the majority of chromaffin cell precursors require axons to navigate into this tissue. However, we also found that 31% of chromaffin cells still navigated into the adrenal primordia after ablation of preganglionic neurons and their axons in *Olig2-Cre; Rosa^DTA^* mice. Possible explanations for this observation are that a small number of preganglionic axons may remain after genetic ablation or that visceral sensory neurons, which also innervate the adrenal primordia (Parker et al., 1993) and which are not ablated in *Olig2-Cre; Rosa^DTA^* mice, may act as an alternative substrate for axon-associated chromaffin cell precursors to enter this tissue.

Our analysis of axonal innervation found that pioneering preganglionic axons enter the adrenal primordia between E11.0 and E11.5, when they split from the gonads. NRP1 and NRP2 are co-expressed on preganglionic axons, and their ligands are expressed in a spatiotemporal pattern consistent with guiding these axons away from the gonads and into the regions of the adrenal anlagen. In particular, the ventral regions of the urogenital ridge showed the highest expression of *Sema3a* and *Sema3f* at the time when axons innervate the adrenal primordia. In agreement with these expression patterns, mice lacking NRP2, and mice lacking SEMA3A and SEMA3F, show ectopic innervation of the gonads.

Previous work in chick has shown that SDF1 and NRG1 are expressed in and around the region of the adrenal primordia and are required for chromaffin cell precursors to colonise the adrenal medulla (Saito et al., 2012). Although the expression pattern of the NRG1 receptor ERBB3 is consistent with a role in guiding preganglionic axons toward the adrenal primordia, our analysis of *Erbb3^−/−^* mice demonstrates that this receptor is not necessary for axonal guidance into this tissue. Future work should therefore examine whether other members of the NRG receptor family are instead important for guiding preganglionic axons that innervate this tissue, and whether SDF signalling plays a complementary or alternative role.

Our findings also provide insight into the mechanisms that promote the physical association between axons and chromaffin cell precursors. Thus, *Wnt1Cre; Nrp1^fl/fl^* embryos, which lack *Nrp1* specifically in NCCs, had ectopically positioned chromaffin cells that lacked the normal association with preganglionic axons. Although this defect is mild, these results suggest that SEMA3A/NRP1 signalling may promote interactions between both cell types, as previously shown for neuregulin/ErbB3 in other regions of the body (Jessen and Mirsky, 2005).

Taken together, our study demonstrates that preganglionic axons serve as anatomical scaffolds to direct chromaffin cell precursors into the adrenal primordia. This mechanism, in which central nervous system-derived axons regulate the formation of their target tissue, is analogous to the mechanisms controlling formation of the parasympathetic ganglia in the head (Coppola et al., 2010; Dyachuk et al., 2014; Espinosa-Medina et al., 2014, 2016). Thus, rather than being restricted to parasympathetic neurons, this developmental principle appears to be adopted by multiple divisions of the autonomic nervous system.

## MATERIALS AND METHODS

### Mice

All animal experiments were carried out in accordance with ethical guidelines of the SA Pathology Animal Ethics Committee or the UCL Institute of Ophthalmology with a licence from the UK Home Office. To obtain embryos of defined gestational ages, mice were mated in the evening, and the morning of vaginal plug formation was counted as embryonic day (E) 0.5. To lineage trace NCCs and their derivatives, we crossed *Wnt1-Cre* mice to *Z/EG* mice (Novak et al., 2000). To ablate motoneurons in the ventral spinal cord, we mated *Olig2Cre* males to *ROSA^DTA/DTA^* females (Wu et al., 2006). To delete NRP1 specifically in NCCs, we mated *Nrp1^+/−^* males carrying a *Wnt1-Cre* transgene to *Nrp1^fl/fl^* mice (Jiang et al., 2002; Gu et al., 2003). Mice carrying the *Erbb3^−/−^*, *Nrp1^sema+/−^*, *Nrp2^+/−^*, *Sema3a^+/−^* and *Sema3f^+/−^* alleles have been previously described (Giger et al., 2000; Kitsukawa et al., 1997; Sahay et al., 2003; Taniguchi et al., 1997; Gu et al., 2003; Britsch et al., 1998).

### *In situ* hybridisation

Section *in situ* hybridisation was performed as described previously (Schwarz et al., 2004). Riboprobes were transcribed from plasmids containing the cDNA sequence for *Sema3a* and *Sema3f* (Maden et al., 2012).

### Immunohistochemistry

Embryos were fixed in 4% formaldehyde in PBS. All sections were cut at 18 µm on a CM1850 cryostat (Leica) and air-dried for 60 min before immunolabelling. For immunolabelling, cryosections were blocked in 0.2% BSA and 0.5% Triton X-100 in PBS, and stained using the indicated primary antibodies. GFP^+^ NCCs grown on fibronectin-coated slides were fixed in 4% formaldehyde in PBS for 10 min, blocked in 10% DAKO block, 0.5% Triton X-100 in PBS and stained with the indicated primary antibodies. Antibodies used were: rabbit anti-TH (Millipore), 1:300; goat anti-NRP1 (R&D), 1:500; rabbit anti-NRP1 (Abcam), 1:500; rabbit anti-NRP2 (CST), 1:500; mouse anti-TUJ1 (Sigma Aldrich), 1:750; chicken anti-GFP, 1:1000 (Abcam); goat anti-SOX10 (Santa Cruz), 1:200; and rabbit anti-VAChT (Sy Sy), 1:1000. Cryosections and cells were mounted in Prolong Gold antifade reagent with DAPI (Molecular Probes). Confocal images were acquired on a LSM 700 (Zeiss) system. All images were prepared with Adobe Photoshop (Adobe).

### Quantitation of chromaffin cells and NCCs dissociated from axons

Transverse sections spanning the anterior to posterior limits of the adrenal glands of E12.5 and E14.5 embryos were collected in a 1/3 series. The total number of TH^+^ cells within the adrenal glands were manually counted from 20× images from each section spanning the limits of the adrenal gland. Data are represented as the number of TH^+^ cells/section compared with control embryos. SOX10^+^ NCCs dissociated from axons were manually counted from 20× images from each section spanning the limits of the adrenal gland. Data are represented as the number of SOX10^+^ NCCs dissociated from axons/section. SOX10^+^ NCCs dissociated from axons around the adrenal gland of E11.5 embryos were manually counted from whole-mount images. Data are presented as the total number of SOX10^+^ NCCs dissociated from axons/embryo.

### Isolation and culture of primary chromaffin cell precursors

Primary NCCs and chromaffin cell precursors were isolated from E11.5 *Wnt1Cre; Z/EG* embryos as previously described (Lumb et al., 2014). The aorta-gonadal-mesonephros region ventral to the dorsal aorta was dissected away from all other tissue and dissociated using Tryple Express (Invitrogen) for 30 min at room temperature. Dissociated cells were centrifuged, resuspended in NCC growth media (DMEM, 5% chicken embryo extract, 10 mM HEPES, 2% B27, 1%N2, 20 ng/ml IGF and 100 U/ml P/S) and plated in eight-well dishes coated with 50 mg/ml fibronectin (Roche) at a concentration of 5×10^4^ cells/well. After 12 h, cells were fixed in 4% PFA for immunolabelling or used for functional assays.

Recombinant purified SEMA3A was prepared as described previously (Ito et al., 2000). Briefly, conditioned medium was harvested from CHO cells transiently transfected with a Sema3A-His expression vector for 3 days. Conditioned medium was applied to 1 ml Protino Ni-NTA resin (Macherey-Nagel). The column was washed with 20 ml of 10 and 50 mM imidazole (pH 8.0) and SEMA3A eluted in 250 mM imidazole (pH 8.0). Fractions containing SEMA3A were dialysed with PBS and concentrated with Vivaspin 500 50 kDa ultrafiltration columns (GE Healthcare). SEMA3A activity was assayed on E12.5 mouse dorsal root ganglia with one unit being defined as the amount required to collapse 50% of the growth cones. One unit of SEMA3A (approximately 5 μg/ml) or BSA (final concentration of 0.0125%) was added to GFP^+^ NCCs. Images were taken every 20 s at 20× magnification on a CV1000 (Olympus) for 10 min pre- and post addition of SEMA3A or BSA.

### DiI labelling

E11.5 wild-type embryos were fixed for 1 h in 4% formaldehyde in PBS and sectioned at a thickness of 300 µm on a LEICAVT1200 vibratome (Leica). DiI crystals (Invitrogen) were then placed into small incisions in the adrenal primordia. DiI-implanted sections were incubated in PBS with 0.02% sodium azide at 37°C in the dark for 2 days. Images were acquired on a LSM 700 (Zeiss) system as described above.

### Statistical analysis

All data are presented as mean±s.e.m. and analysed using two-tailed Student's *t*-test. In all studies, *P*<0.05 was considered to be statistically significant. *n* refers to the number of individual embryos used for analysis, which were obtained from at least four separate litters.

## Supplementary Material

Supplementary information
