# Supplementary material for: Neuropilins guide preganglionic sympathetic axons and chromaffin cell precursors to establish the adrenal medulla
Source: Development. 2018 Nov 2;145(21):dev162552. doi: 10.1242/dev.162552 (PMC6240312; doi:10.1242/dev.162552)
Supplement: Supplementary information [file develop-145-162552-s1.pdf]

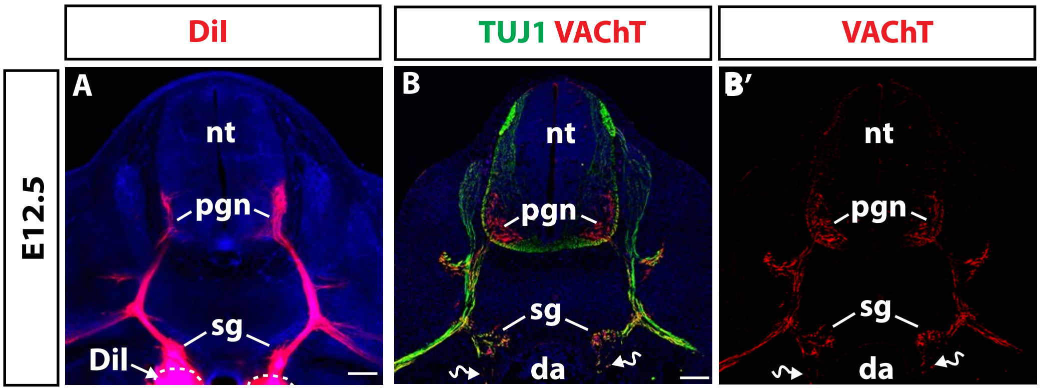

Fig. S1. Identification of preganglionic neurons innervating the adrenal primordia. (A) DiI crystals placed into the adrenal primordia (dashed lines) of wild type E12.5 embryos trace the axons of preganglionic neurons (pgn) back to the intermediolateral motor columns of the neural tube. (B-B') Immunolabelling for TUJ1 and VAChT on an E12.5 transverse section (from an embryo age-matched to that in A) shows the navigation path of preganglionic axons from the intermediolateral motor columns to the sympathetic ganglia and further ventrally toward the adrenal primordia (curved arrow). Blue, DAPI. Scale bars = 100 µm.

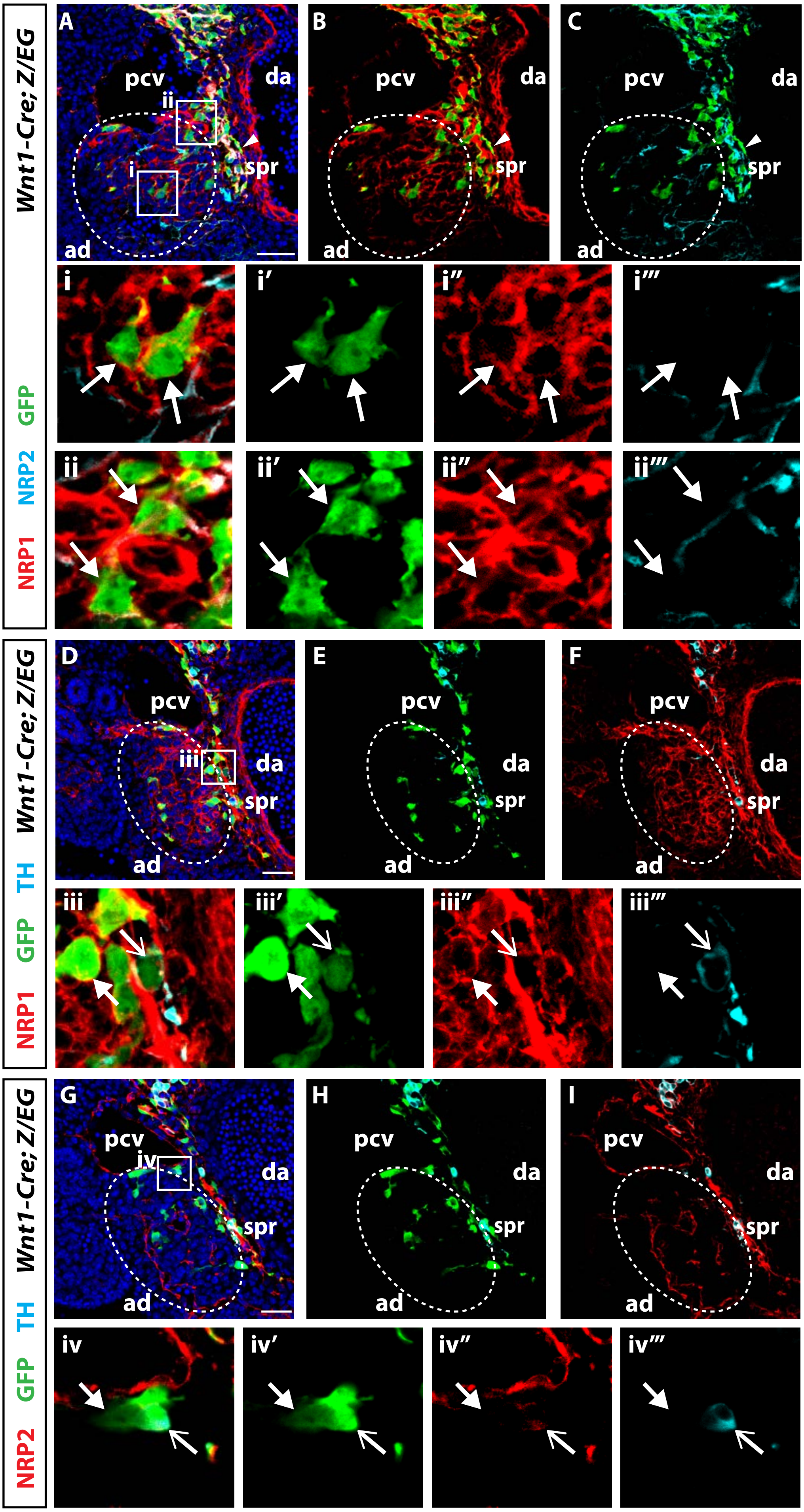

Wnt1-Cre; Z/EG
GFP NRP2 NRP1
A B C
pcv da spr ad
i ii
i i' i'' i'''
ii ii' ii'' ii'''
Wnt1-Cre; Z/EG
TH GFP NRP1
D E F
pcv da spr ad
iii
iii iii' iii'' iii'''
Wnt1-Cre; Z/EG
TH GFP NRP2
G H I
pcv da spr ad
iv
iv iv' iv'' iv'''

Fig. S2. NRP1, but not NRP2, is expressed in sympathoadrenal NCCs. (A-I) Transverse sections through the adrenal primordia (ad, white dashed circle) of E11.5 *Wnt1-Cre; Z/EG* embryos, immunolabelled for GFP, TH, NRP1 and NRP2. (A-C) NRP1 is expressed in all NCCs within the adrenal primordia (i'', white arrows), in NCCs along the migration path to the adrenal primordia between the dorsal aorta (da) and posterior cardinal vein (pcv; ii'', white arrows), and in NCCs in the suprarenal ganglia (spr). NRP1 is also expressed in adrenal primordial tissue, dorsal aorta and axons innervating past the dorsal aorta (B, white arrow head). In contrast, NRP2 is not expressed in NCCs within the adrenal primordia (i''') or along the migration path to the adrenal primordia and (ii'''). NRP2 labels blood vessels and axons within the primordia as well as axons innervating past the dorsal aorta (C, white arrow head). (D-F) NRP1 is expressed by all NCCs regardless of their expression of TH (iii-iii''', arrow represents undifferentiated NCC and open arrow represents differentiated NCC). (G-I) A transverse section adjacent to that shown in (D) demonstrates that NRP2 is expressed in NCCs only after they commence expression of TH (iv-iv''', open arrow) and not in NCCs lacking TH (iv-iv''', arrow). Blue, DAPI. Scale bars = 50µm.

Development • Supplementary information

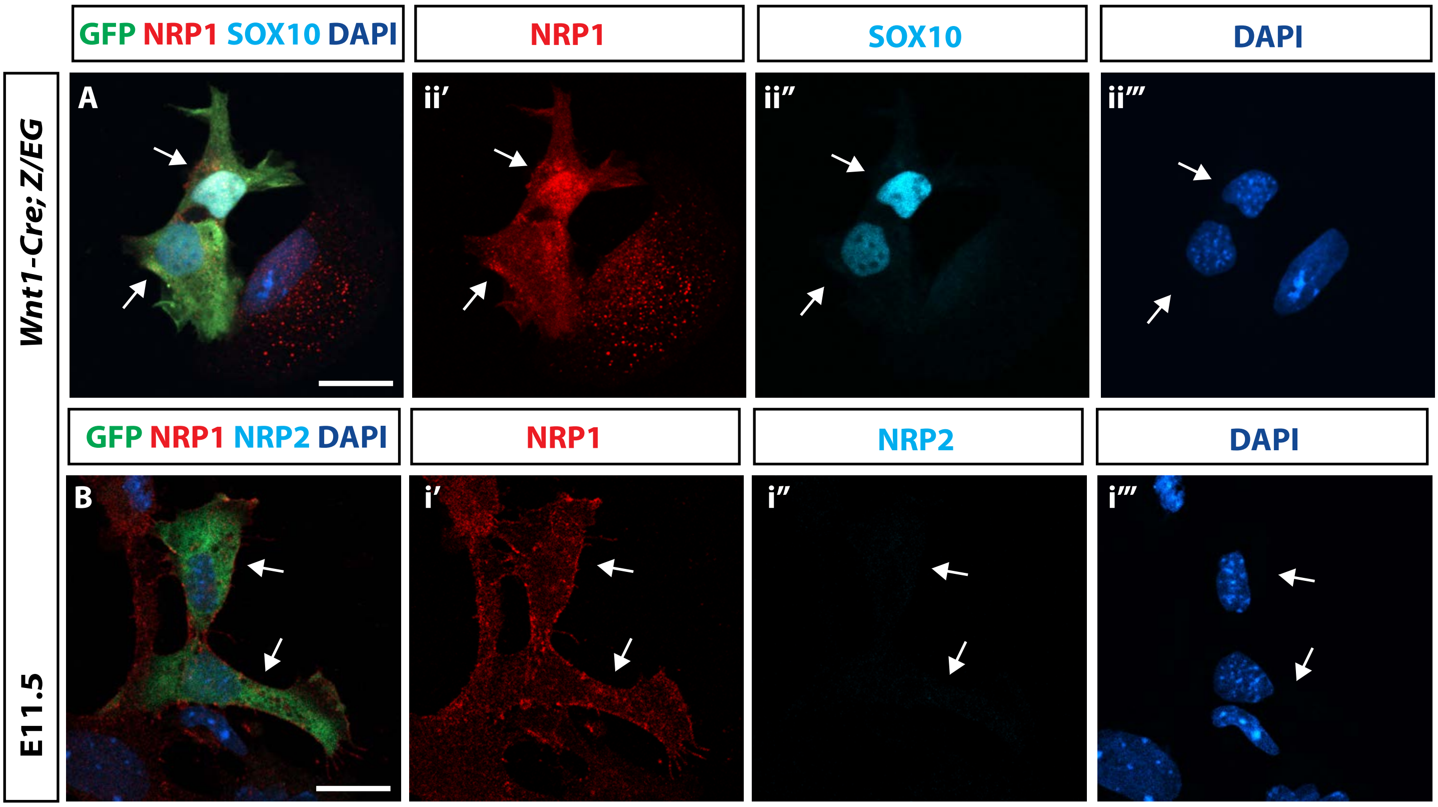

Fig. S3. NRP1 is expressed on the surface of all sympathoadrenal NCCs. (A-B) GFP^{+} sympathoadrenal NCCs isolated from *Wnt1-Cre; Z/EG* embryos immunolabelled for GFP, NRP1, NRP2 and SOX10. All GFP^{+} / SOX10^{+} sympathoadrenal NCCs express NRP1 (A-B) (white arrows) but lack NRP2 (B). Blue, DAPI. Scale bar = 20µm.

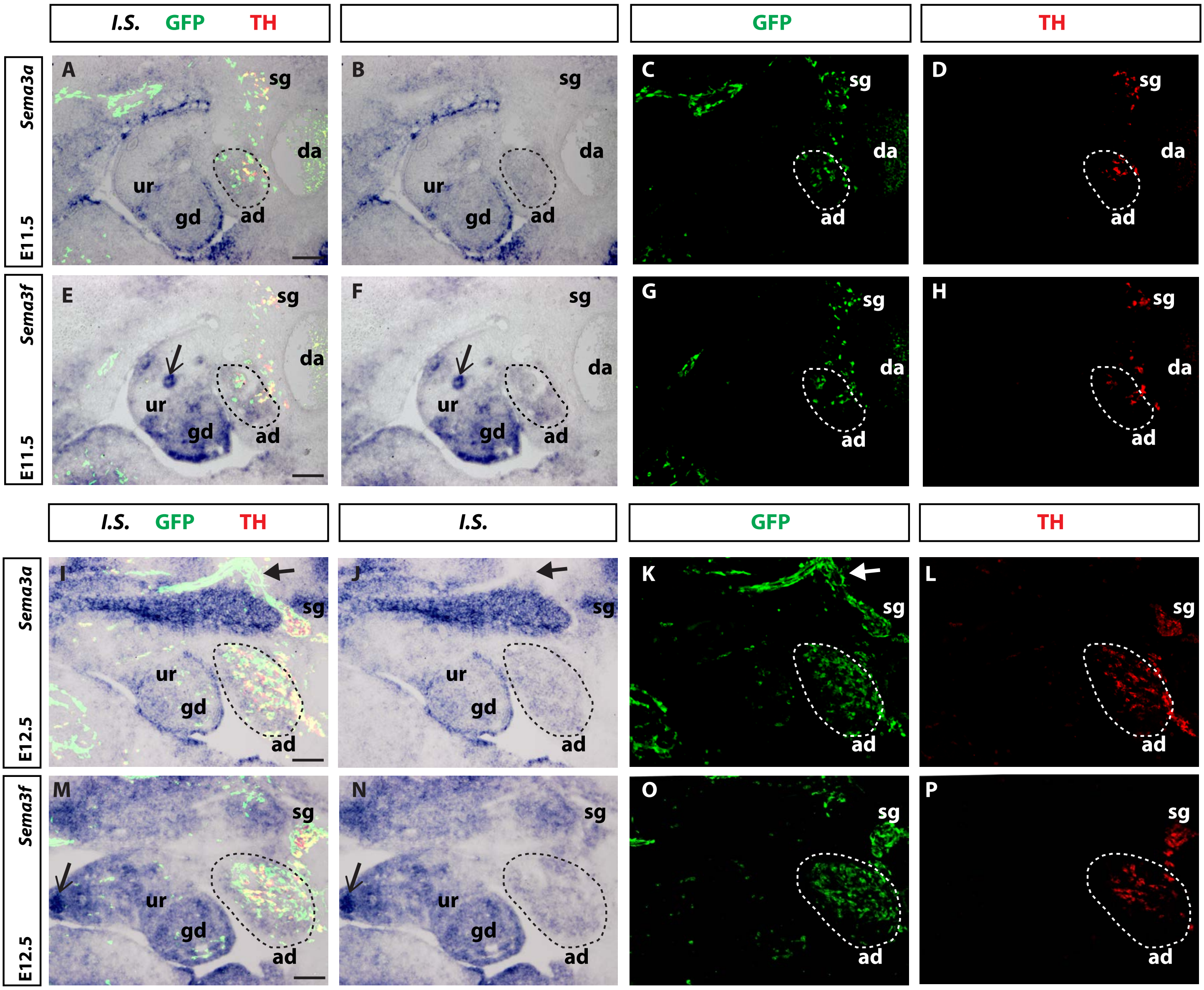

Fig. S4. Class 3 Semaphorins define guidance paths for NCCs / chromaffin cell precursors and axons. (A-P) *in situ* hybridisation of *Sema3a* and *Sema3f* on transverse serial sections of E11.5 and E12.5 *Wnt1-Cre; Z/EG* embryos immunostained for GFP and TH. *Sema3a* is expressed within the urogenital ridge (ur) at E11.5 (A-B) and E12.5 (I-J), with strongest expression within the ventral gonadal tissue (gd) and epithelial lining. *Sema3a* is also expressed diffusely through the adrenal primordia (ad) and in connective tissue surrounding bundles of axons innervating the sympathetic ganglia (sg) (arrow, I-J). *Sema3f* is expressed in the ventral gonadal tissue and in tubules of the metanephros (open arrow) at E11.5 and E12.5 and throughout the urogenital ridge at E12.5 (M-N). Scale bars = 100μm.

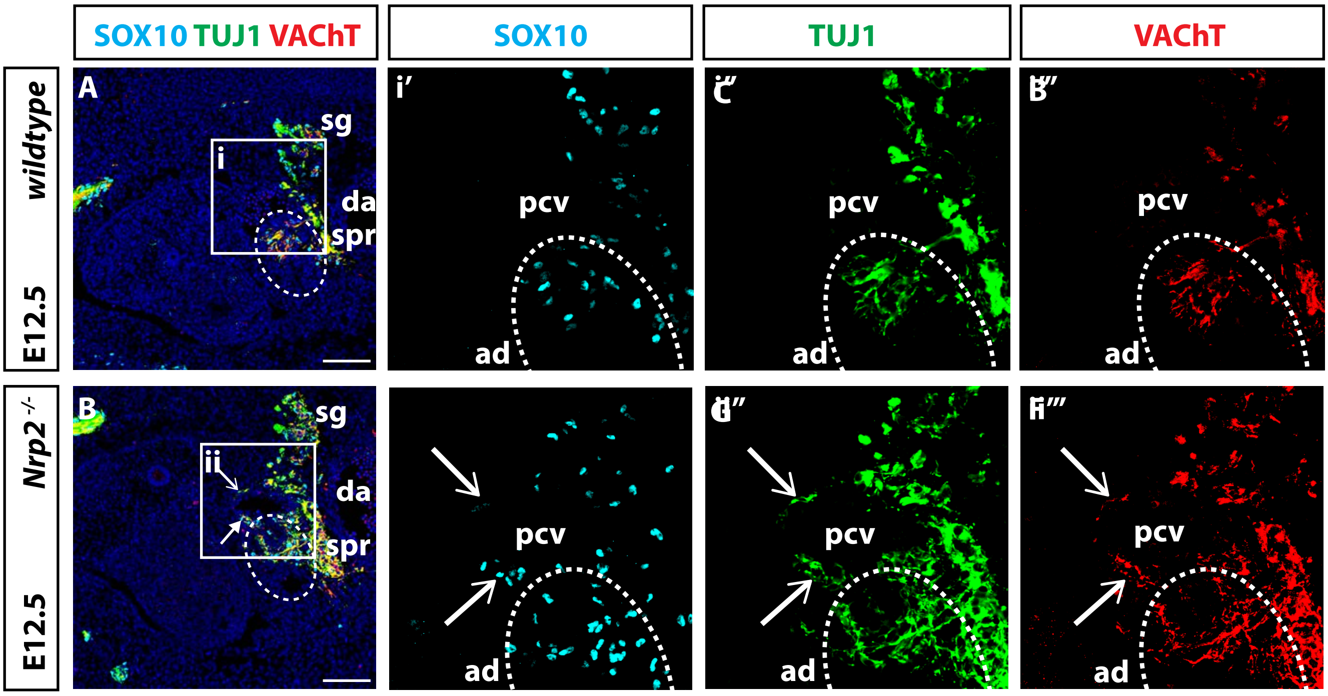

Fig S5. NRP2 guides preganglionic axons into the adrenal primordia.

(A-B) Serial transverse sections through the adrenal primordia (ad) of the E12.5 *Nrp2*$^{+/+}$ (A) and *Nrp2*$^{-/-}$ (B) embryos shown in Fig. 4, immunolabelled for SOX10, TUJ1 and VAChT. Axons aberrantly navigate around the posterior cardinal vein, with SOX10^{+} NCCs in tight association. sg, sympathetic ganglia; da, dorsal aorta; pcv, posterior cardinal vein; Blue, DAPI; n=5 / genotype. Scale bar = 100µm.

Development • Supplementary information

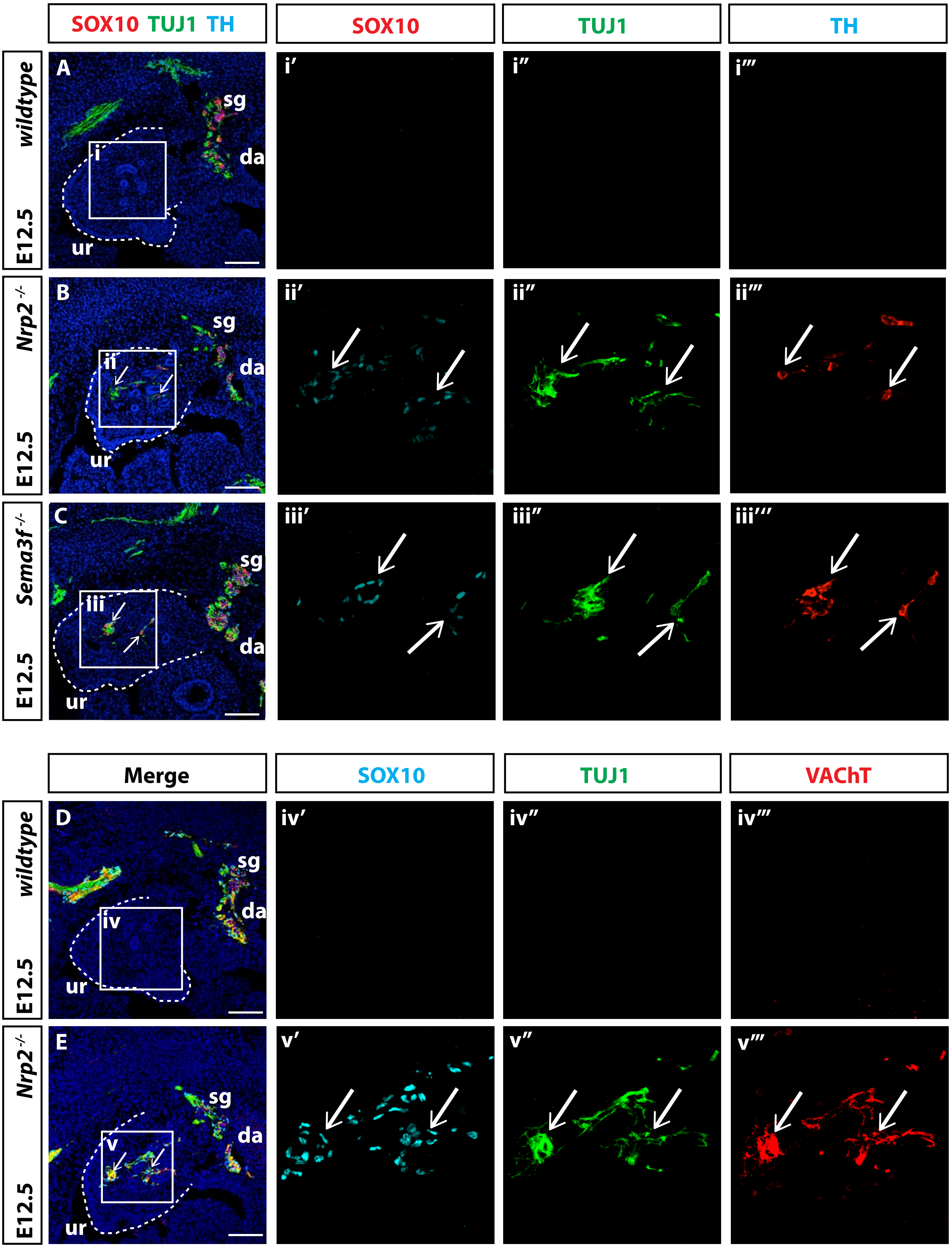

Development • Supplementary information

Fig S6. SEMA3F / NRP2 signalling inhibits preganglionic innervation of the urogenital ridge. Transverse sections through the urogenital ridge (ur) of E12.5 control (A, D; *Nrp2*$^{+/+}$), *Nrp2*$^{-/-}$ (B, E) and *Sema3f*$^{-/-}$ (C) embryos immunolabelled for SOX10, TUJ1, TH and VAChT. (A-C) Axons aberrantly enter the urogenital ridge of are *Nrp2*$^{-/-}$ and *Sema3f*$^{-/-}$ embryos and are aligned with SOX10^{+} NCCs and TH^{+}/ TUJ1^{low} chromaffin cells. (D-E) Serial transverse sections to those shown in (A-B) have ectopic VAChT^{+} axons within the urogenital ridge. sg, sympathetic ganglia; da, dorsal aorta; Blue, DAPI; n=5 / genotype. Scale bars = 100µm.

Development • Supplementary information

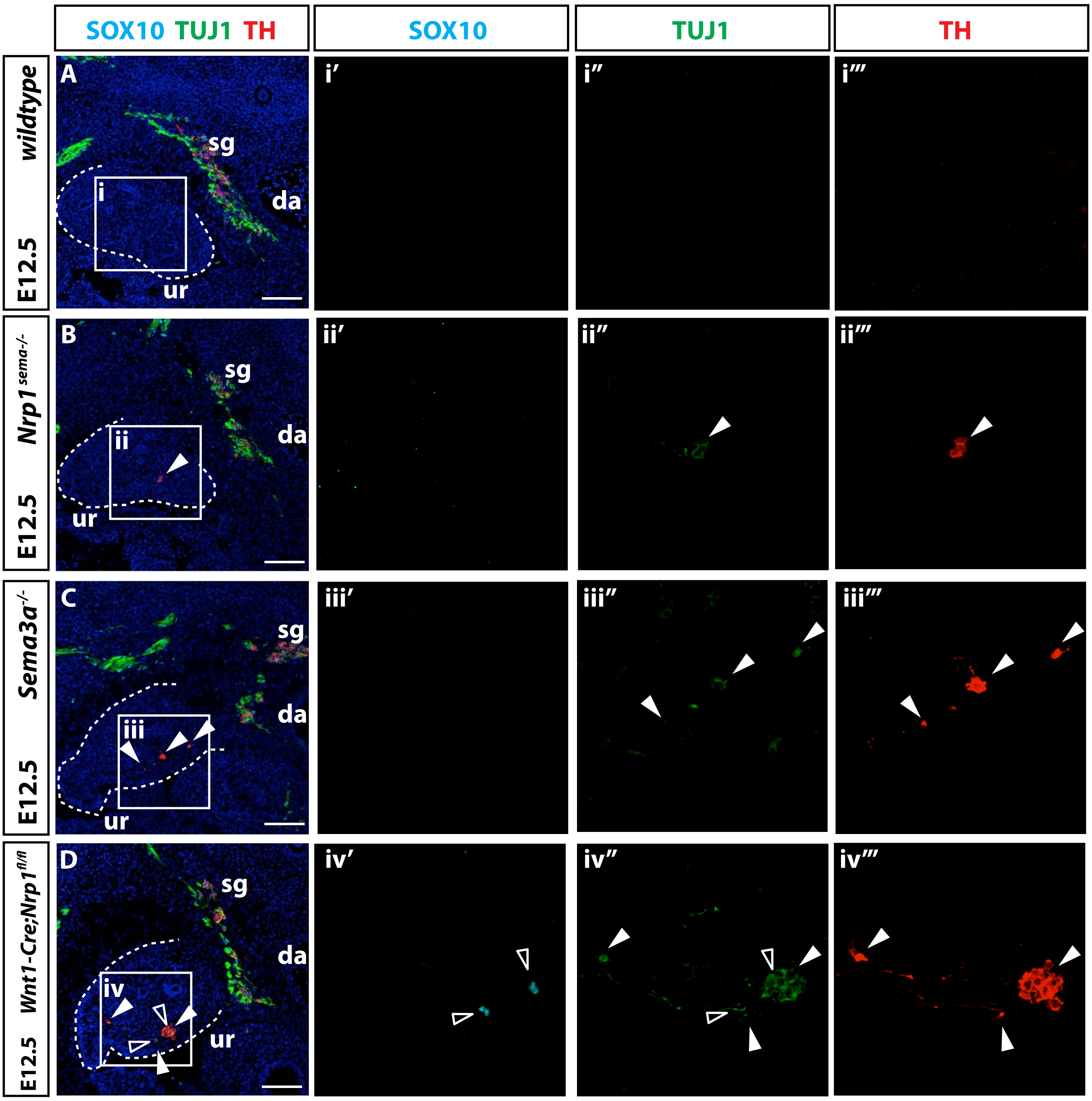

Fig. S7. SEMA3A / NRP1 signalling inhibits NCC migration into the urogenital ridge. Transverse sections through the urogenital ridge (ur) of E12.5 control (A), *Nrp1*$^{sema-/-}$ (B), *Sema3a*$^{-/-}$ (C) and *Wnt1-Cre; Nrp1*$^{fl/fl}$ (D) embryos immunolabelled for SOX10, TUJ1 and TH. Arrowheads indicate ectopic TH^{+} / TUJ1^{low} chromaffin cells within the urogenital ridge in the absence of axons, and open arrowheads indicate ectopic SOX10^{+} chromaffin cell precursors in the absence of axons. da, dorsal aorta; sg, sympathetic ganglia; blue, DAPI; n=5 / genotype. Scale bars = 100µm.

Development • Supplementary information

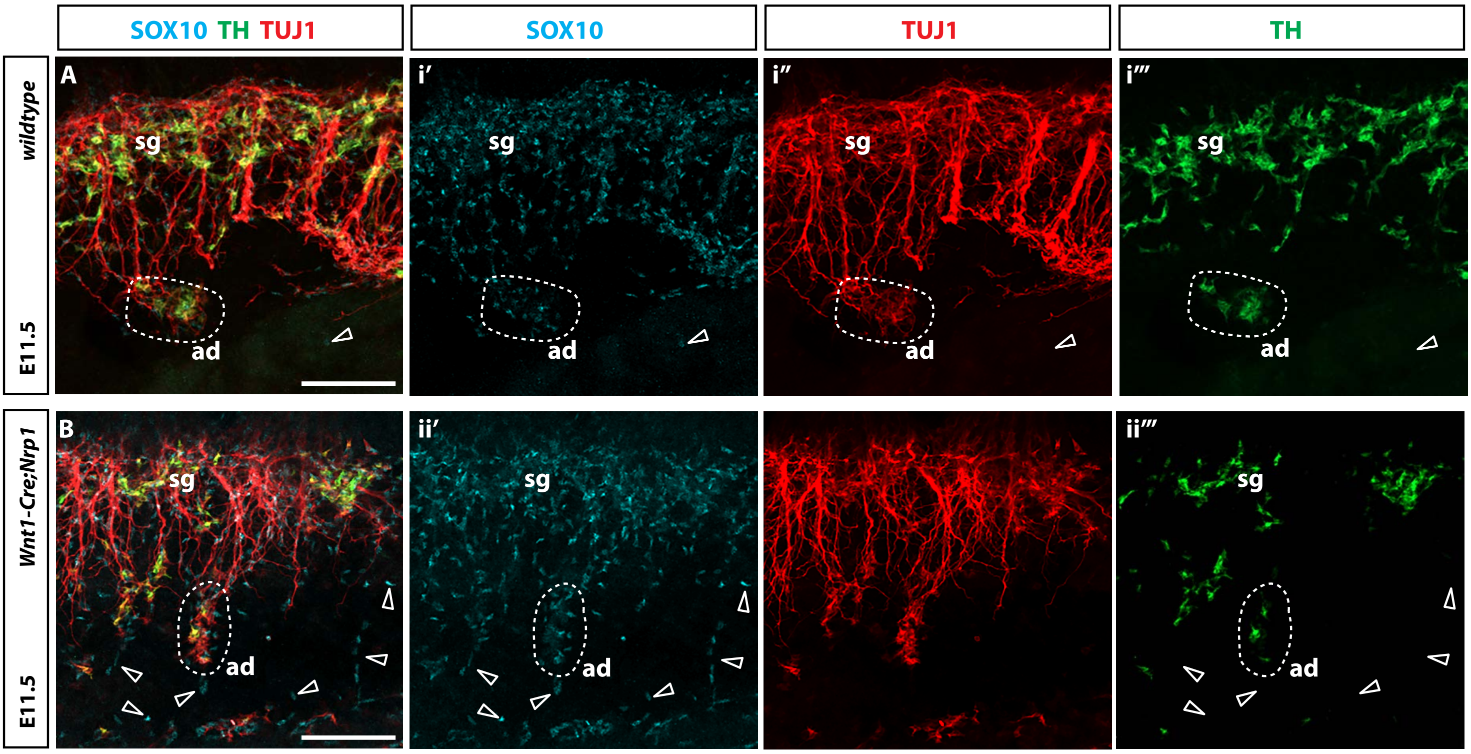

Fig. S8. NRP1 regulates the association of SOX10^{+} cells with axons. Whole E11.5 control (A) and *Wnt1-Cre; Nrp1$^{fl/fl}$* (B) embryos immunolabelled for SOX10, TUJ1 and TH. (B) SOX10^{+} cells are dissociated from axons around the region of the adrenal primordia (ad, open arrowheads) of *Wnt1-Cre; Nrp1$^{fl/fl}$* embryos. sg, sympathetic ganglia; Blue, DAPI; n=5 / genotype. Scale bars = 100µm.

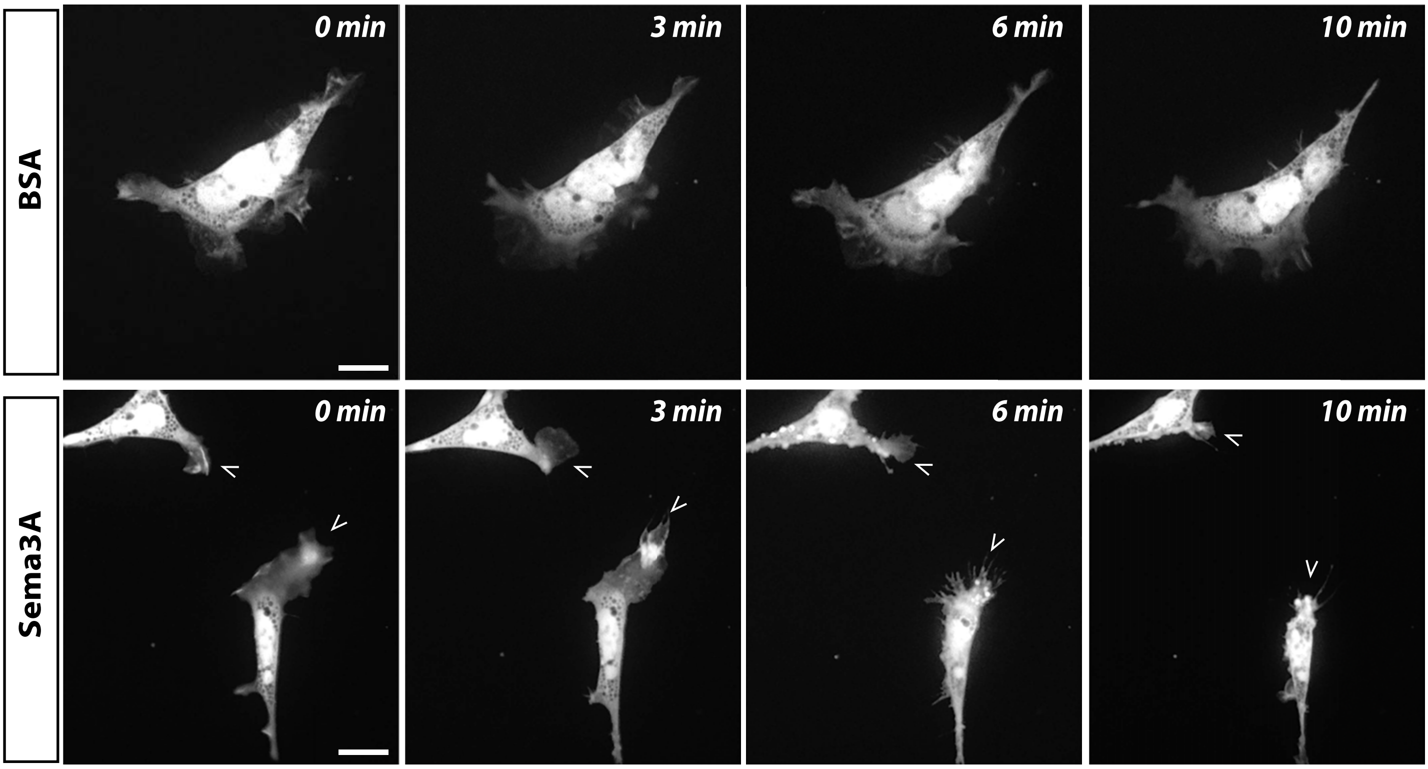

Fig. S9. SEMA3A collapses sympathoadrenal NCCs. Time-lapse sequence of GFP^+ sympathoadrenal NCCs isolated from *Wnt1-Cre; Z/EG* embryos after treatment with BSA or SEMA3A. (B) Sympathoadrenal NCCs retract their protrusions (open arrowhead) and have collapsed within 10 min after addition of SEMA3A. Scale bar = 20µm.

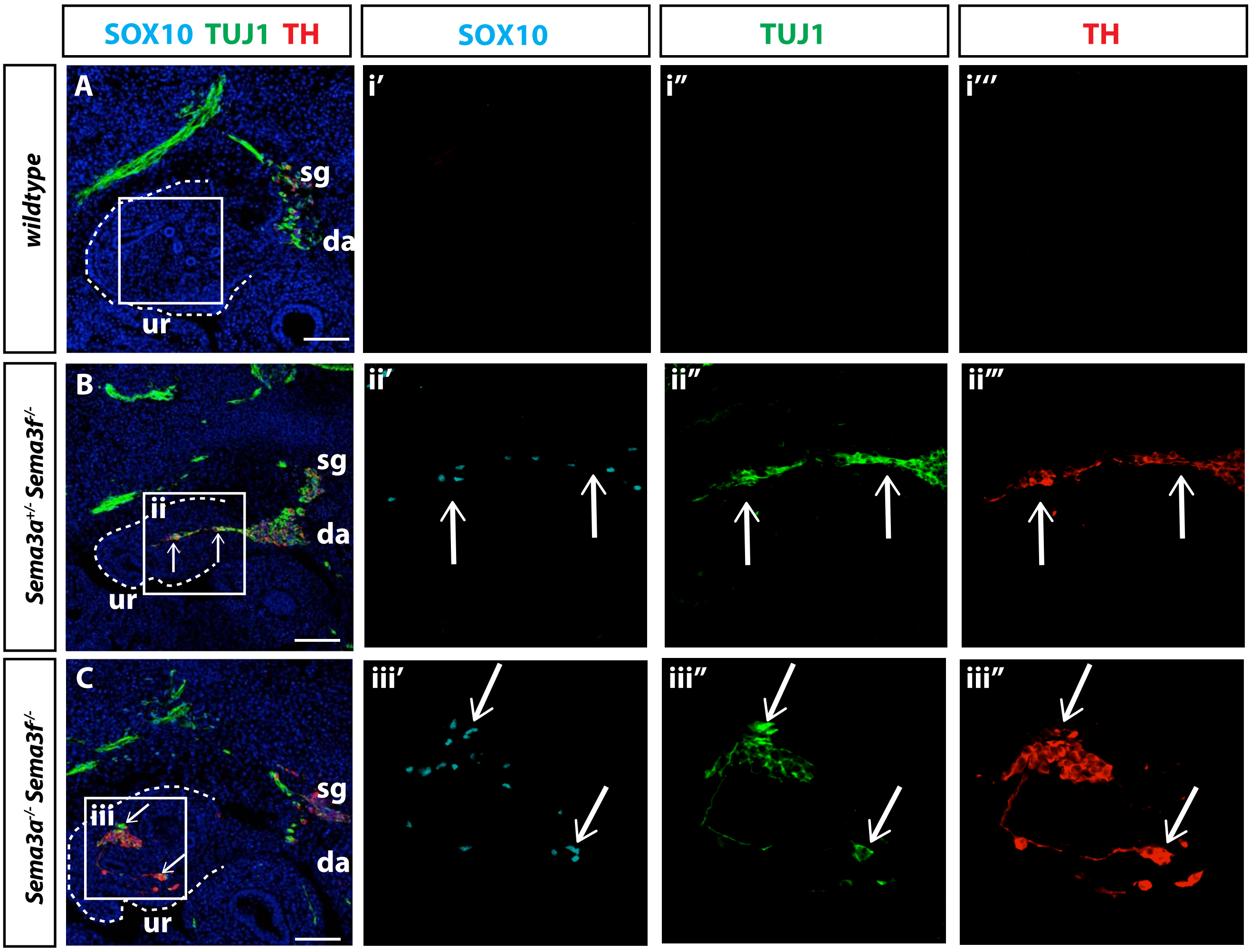

Fig. S10. NRPs cooperate to guide preganglionic axons and NCCs into the adrenal primordia.

Transverse sections through the urogenital ridge (ur) of E12.5 *Sema3a*$^{+/+}$; *Sema3f*$^{+/+}$ (A), *Sema3a*$^{+/-}$; *Sema3f*$^{-/-}$ (B) and *Sema3a*$^{-/-}$; *Sema3f*$^{-/-}$ (C) embryos immunolabelled for SOX10, TUJ1 and TH. (B) A large number of axons, SOX10^{+} NCCs and TH^{+} / TUJ1^{low} chromaffin cells are ectopically positioned in the urogenital ridge (open arrow) of *Sema3a*$^{-/-}$; *Sema3f*$^{+/-}$ embryos. (C) The number of ectopic axons and chromaffin cells within the urogenital ridge (open arrow) is compounded in *Sema3a*$^{-/-}$; *Sema3f*$^{-/-}$ embryos (open arrows) ; n=4 / genotype. Blue, DAPI. Scale bars = 100µm.

Development • Supplementary information
